# Supplementary material for: Identifying membrane-bound transcriptional regulatory proteins from rare but evolutionarily conserved domain combinations
Source: bioRxiv. 2025 Dec 21:2025.12.20.695554. Preprint. [Version 1] doi: 10.64898/2025.12.20.695554 (PMC12724719; doi:10.64898/2025.12.20.695554)
Supplement: 1 [file NIHPP2025.12.20.695554V1-supplement-1.pdf]

bioRxiv preprint doi: https://doi.org/10.64898/2025.12.20.695554; this version posted December 21, 2025. The copyright holder for this preprint (which was not certified by peer review) is the author/funder, who has granted bioRxiv a license to display the preprint in perpetuity. It is made available under aCC-BY 4.0 International license.

Supplementary Figures

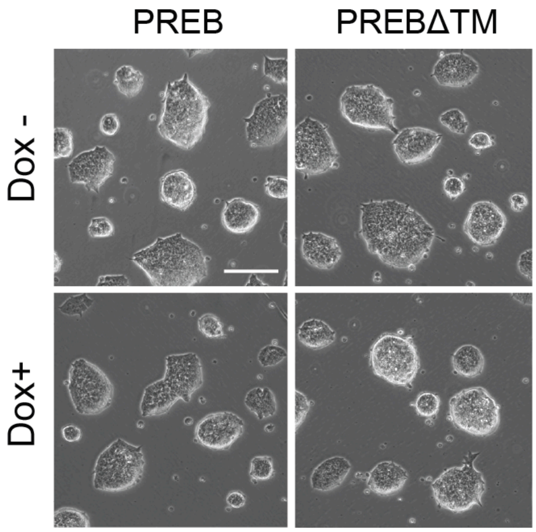

Supplementary Figure S1. Overexpression of PREB does not change the morphology of mouse ES cells

Brightfield images of mouse ES cells expressing full-length PREB and PREBΔTM following treatment with 0.5 µg/mL of Dox for 24 hours (Dox+). Untreated cells (Dox-) were used as controls (Scale bar: 100 µm).

bioRxiv preprint doi: https://doi.org/10.64898/2025.12.20.695554; this version posted December 21, 2025. The copyright holder for this preprint (which was not certified by peer review) is the author/funder, who has granted bioRxiv a license to display the preprint in perpetuity. It is made available under aCC-BY 4.0 International license.

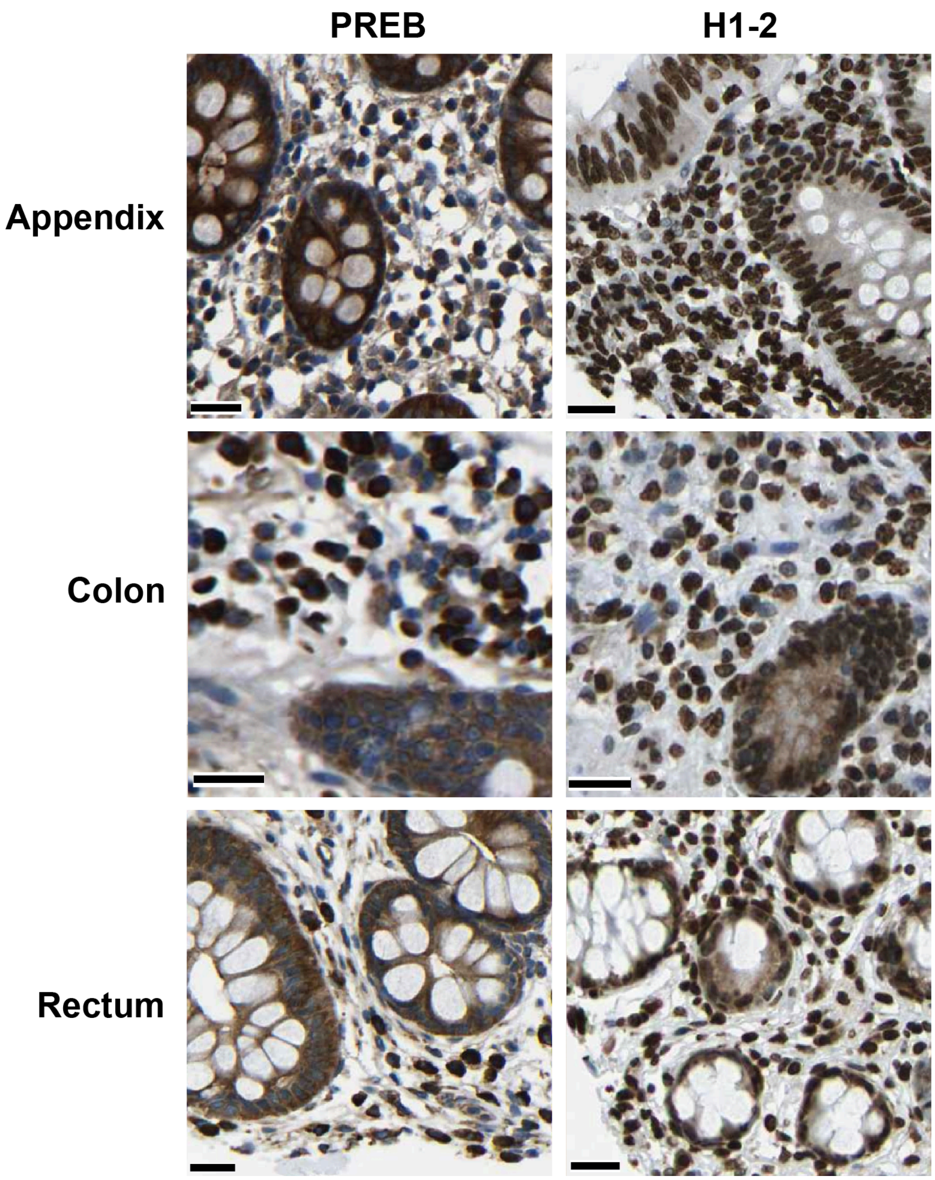

Supplementary Figure S2. Endogenous PREB localizes to the nucleus in cells of the human appendix, colon, and rectum.

Immunohistochemistry-based protein expression profiles of PREB and H1-2 (H1.2 linker histone) in human appendix, colon, and rectum tissues (scale bar = 25 μm), showing data from the Human Protein Atlas (63). The brown 3,3'-diaminobenzidine staining labels target proteins; the blue hematoxylin preferentially labels cell nuclei. These three profiles suggest nuclear localization of PREB in many cells in tissue sections of human appendix, colon, and rectum, but with some variation in localization across specific cell types. In comparison, the histone H1-2 exhibits nuclear localization in almost all cells in tissue-matched sections.

bioRxiv preprint doi: https://doi.org/10.64898/2025.12.20.695554; this version posted December 21, 2025. The copyright holder for this preprint (which was not certified by peer review) is the author/funder, who has granted bioRxiv a license to display the preprint in perpetuity. It is made available under aCC-BY 4.0 International license.

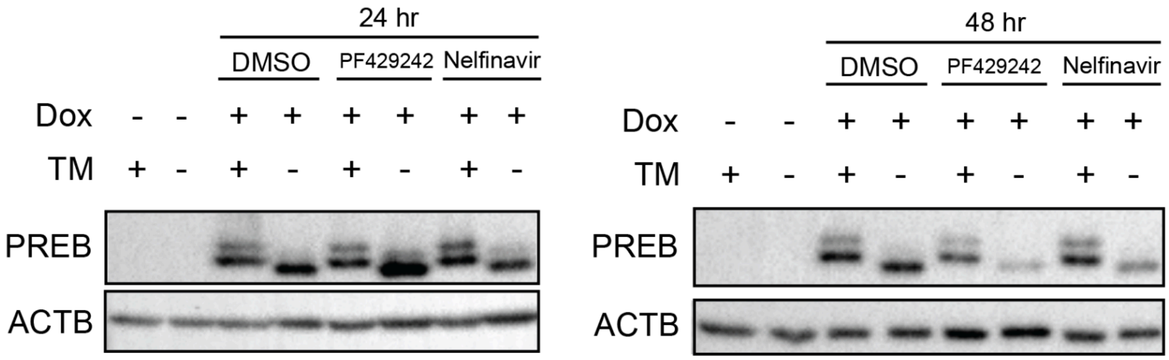

Supplementary Figure S3. S1P and S2P inhibitors do not block the cleavage of PREB

Western blot results after applying S1P and S2P inhibitors for 24 and 48 hours. PREB clones retain two bands after inhibitor treatment, indicating that the two bands do not derive from S1P or S2P protease cleavage activity.

bioRxiv preprint doi: https://doi.org/10.64898/2025.12.20.695554; this version posted December 21, 2025. The copyright holder for this preprint (which was not certified by peer review) is the author/funder, who has granted bioRxiv a license to display the preprint in perpetuity. It is made available under aCC-BY 4.0 International license.

Supplementary Tables

Supplementary Table S1. Primers used for cloning

PREB/PREBΔTM Forward Primer	GACTAGCGGCCGCAATGGGTCGGCGCCGGGGTGTG
PREB Reverse Primer	GGTGCTAGCTTAGAGAAATCCCGGGAAGGC
PREBΔTM Reverse Primer	GGTGCTAGCTTAACTCCGCCGTGAAGGCAG

Supplementary Table S2. Selected 855 [domain, orthogroup] pairs and corresponding proteins

Supplementary Table 2 is available for download at https://doi.org/10.5281/zenodo.17351618.
